# Microbiological quality assessment of fresh produce: Potential health risk to children and urgent need for improved food safety in school feeding schemes

**DOI:** 10.1002/fsn3.3506

**Published:** 2023-07-09

**Authors:** Thabang Msimango, Stacey Duvenage, Erika M. Du Plessis, Lise Korsten

**Affiliations:** ^1^ Department of Plant and Soil Sciences University of Pretoria Pretoria South Africa; ^2^ Department of Science and Innovation‐National Research Foundation Centre of Excellence in Food Security Pretoria South Africa; ^3^ Natural Resources Institute, Faculty of Engineering and Science University of Greenwich London UK

**Keywords:** fresh produce, microbiological quality, potential health risk, school feeding, school‐going children

## Abstract

About 388 million school‐going children worldwide benefit from school feeding schemes, which make use of fresh produce to prepare meals. Fresh produce including leafy greens and other vegetables were served at 37% and 31% of school feeding programs, respectively, in Africa. This study aimed at assessing the microbiological quality of fresh produce grown onsite or supplied to South African schools that are part of the national school feeding programs that benefit over 9 million school‐going children. Coliforms, *Escherichia coli*, Enterobacteriaceae, and *Staphylococcus aureus* were enumerated from fresh produce (*n* = 321) samples. The occurrence of *E. coli*, *Listeria monocytogenes*, *Salmonella* spp., and extended‐spectrum β‐lactamase (ESBL)‐producing Enterobacteriaceae was determined. Presumptive pathogens were tested for antimicrobial resistance. *E. coli* was further tested for diarrheagenic virulence genes. Enterobacteriaceae on 62.5% of fresh produce samples (200/321) exceeded previous microbiological guidelines for ready‐to‐eat food, while 86% (276/321 samples) and 31.6% (101/321 samples) exceeded coliform and *E. coli* criteria, respectively. A total of 76 Enterobacteriaceae were isolated from fresh produce including *E. coli* (*n* = 43), *Enterobacter* spp. (*n* = 15), and *Klebsiella* spp. (*n* = 18). Extended‐spectrum β‐lactamase production was confirmed in 11 *E. coli*, 13 *Enterobacter* spp., and 17 *Klebsiella* spp. isolates. No diarrheagenic virulence genes were detected in *E. coli* isolates. However, multidrug resistance (MDR) was found in 60.5% (26/43) of the *E. coli* isolates, while all (100%; *n* = 41) of the confirmed ESBL and AmpC Enterobacteriaceae showed MDR. Our study indicates the reality of the potential health risk that contaminated fresh produce may pose to school‐going children, especially with the growing food safety challenges and antimicrobial resistance crisis globally. This also shows that improved food safety approaches to prevent foodborne illness and the spread of foodborne pathogens through the food served by school feeding schemes are necessary.

## Graphical abstract

This study sought to determine the microbiological safety of fresh produce grown onsite or supplied to South African schools as part of the national school feeding program. The microbiological quality of fresh produce grown in school gardens and fresh produce delivered to schools by contracted companies may not always be of good microbiological standards. With the growing food safety and antibiotic resistance crisis globally, the current findings demonstrate the potential health risk to school‐going children.

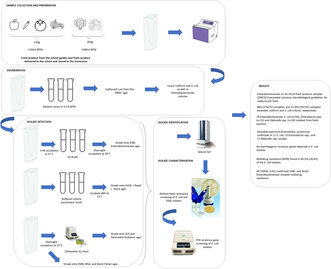

## 1 INTRODUCTION

There are 388 million schoolchildren in 161 countries receiving meals at schools globally (World Food Programme, 2021). The largest school feeding programs are in India (90 million children), Brazil and China (40 million children), the United States of America (30 million children), and Egypt (11 million children; World Food Programme, 2021). South Africa provides meals to over 9.6 million school‐going children (Department of Basic Education, 2019). These school feeding schemes make use of vegetables to prepare meals for the school children. In addition, school children normally also get fruit with their meals (Department of Basic Education, 2019).

Fresh produce is associated with health benefits, and thus a desirable component of any meal (Weichselbaum & Buttriss, 2014). However, fresh produce has also been linked to foodborne disease outbreaks (Park et al., 2012). Globally, an estimated 600 million foodborne disease cases occur every year, resulting in over 400,000 deaths mostly caused by bacterial pathogens (Havelaar et al., 2015; World Health Organization, 2015). *Escherichia coli* are often a harmless commensal organism, however, pathogenic strains cause diarrhea and other serious gastrointestinal diseases (Hamilton et al., 2010). Other major foodborne pathogens include *Salmonella* spp. and *Listeria monocytogenes* (Centers for Disease Control and Prevention, 2020). In addition to these pathogens, extended‐spectrum β‐lactamase (ESBL)‐producing Enterobacteriaceae have also been detected in food linked to foodborne disease outbreaks (Calbo et al., 2011; Lavilla et al., 2008). These ESBL‐ as well as AmpC β‐lactamase‐producing Enterobacteriaceae have also been detected in fresh vegetables (Berner et al., 2015; Blaak et al., 2014; Li et al., 2018; Richter et al., 2019) and are therefore a reason for concern especially with the global drive to increase consumption of fresh produce. Moreover, with increasing antibiotic resistance in bacterial pathogens, general treatment of foodborne diseases is a growing concern in healthcare (Centers for Disease Control and Prevention, 2013; WHO, 2016). In addition to illness, foodborne diseases could result in death and long‐term chronic ailments (Lindsay, 1997). Anxiety, an indirect effect of foodborne diseases, can also exist in communities that have experienced outbreaks and further lack trust in the food system (Bryan, 1978). Foodborne diseases also put extreme pressure on the public health system as well as on healthcare workers (Bryan, 1978). For school‐going children, who are classified as the most vulnerable group (Kirk et al., 2016), foodborne disease also means a loss of learning time and negatively impacts their growth and development (Sibanyoni & Tabit, 2016).

The National Institute for Communicable Diseases (NICD) reported 31 cases of foodborne and/or waterborne disease outbreaks in the first 6 months of 2017 in South Africa (SA), nine of which were recorded from schools (NICD, 2017). Fresh produce was implicated in two of these outbreaks, where *Salmonella* spp. and *Clostridium perfringens* were detected (NICD, 2014; Msomi, 2017).

The safety of fresh produce used to make meals and served at schools, globally, is, therefore, a concern and warrants further investigations. Moreover, as far as the authors are aware, the potential food safety risk associated with fresh produce in schools has not been explored in South Africa. This study investigated the microbiological safety of fresh produce (spinach, Chinese spinach, carrots, cabbage, onions, tomatoes, lettuce, and apples) grown at or supplied to schools to prepare meals.

## 2 METHODS AND MATERIALS

### 2.1 Sample collection

Fresh produce was collected from six schools in Gauteng Province (schools 1–3 in Ekhuruleni district and schools 4–6 in Tshwane district) and six schools in the Mpumalanga Province (schools 7–9 in Nkangala district and schools 10–12 in the Gert Sibande district) after permission was granted by the provincial Departments of Basic Education, each school was visited twice. Fresh produce (growing in gardens on the school premises) available at the time of sampling was collected aseptically at five points in the school garden per crop planted (*n* = 186) and from three different packages in the kitchen storage area per produce item (*n* = 135). Each sample consisted of an equal number of three different fresh produce units. Samples included spinach (Swiss chard), Chinese spinach, lettuce (iceberg), onions, cabbage, apples, tomatoes, and carrots. These samples were transported in cool boxes to the Plant Pathology Laboratories, University of Pretoria, and kept refrigerated (4°C) until processing was done, usually within 48 h.

### 2.2 Microbiological analysis

Fresh produce (50 g of spinach, lettuce, and cabbage, and 150 g of apples, onions, carrots, and tomatoes) was macerated in buffered peptone water (BPW; Merck) [200 mL for spinach, cabbage, and lettuce (1:4 ratio), and 250 mL for apples, tomatoes, onions, and carrots (1:5 ratio)]; (Xu et al., 2015) in Seward stomacher 400 circulator strainer bags (Lasec, Johannesburg), using the Seward Stomacher (Lasec) at 230 g for 5 min. A dilution series of each sample was done using 0.1% BPW and spread plated onto Violet Red Bile Glucose agar (Oxoid, Johannesburg; ISO 21528 and ISO 11133:2014) in duplicate to enumerate Enterobacteriaceae, onto Staph Express Count Plates and *E. coli*/coliform Count Plates (3 M, Johannesburg) to enumerate *Staphylococcus aureus* and *E. coli* and coliforms, respectively. Agar plates and count plates were incubated at 37°C for 24 h.

Fresh produce samples in BPW were incubated at 37°C, following 4 h of incubation, 1 mL was transferred to 9 mL of Enterobacteriaceae enrichment broth (EE Broth; Oxoid), and incubated at 30°C for 24 h. Samples in BPW were then further incubated at 37°C for 24 h. Subsequently, samples in BPW were streaked onto Eosin methylene blue agar (Oxoid) for the detection of *E. coli*, Baird–Parker agar (Merck), and Mannitol salt agar (Thermo Fisher Scientific, Johannesburg) for *S. aureus*. The incubated EE broth was streaked onto chromID ESBL agar (Biomeriuex, Johannesburg) to detect ESBL‐producing Enterobacteriaceae. For the detection of *L. monocytogenes* and other *Listeria* species, 1 mL^−1^ of the overnight incubated sample in BPW was transferred to 9 mL^−1^ of buffered Listeria enrichment broth (Oxoid) and incubated at 37°C for 48 h and then streaked onto Agar *Listeria* according to Ottaviani and Agosti (BioRad, AEC Amersham, Johannesburg) and Rapid L. mono agar (BioRad). *Salmonella* spp. detection was done from samples incubated in BPW using the BioRad iQ check *Salmonella* kit (AEC Amersham), following the manufacturer's instructions (AOAC OMA 2017.06). All presumptive positive isolates were identified using matrix‐assisted laser desorption ionization–time of flight mass spectrometry (MALDI‐TOF) in conjunction with the Bruker MALDI Biotyper software (Bruker, Johannesburg; Standing et al., 2013).

### 2.3 Antimicrobial resistance testing and virulence gene screening of *Escherichia coli* isolates

All 43 *E. coli* isolates were subjected to antimicrobial resistance screening using the Kirby–Bauer disc diffusion method (Bauer et al., 1966). *Escherichia coli* isolates were cultured in brain heart infusion broth (BHI) and plated onto Mueller–Hinton agar plates (Thermo Fisher Scientific). *Escherichia coli* isolates were tested against cefotaxime (30 μg), ciprofloxacin (5 μg), chloramphenicol (30 μg), cephalothin (30 μg), gentamicin (100 μg), nitrofurantoin (300 μg), streptomycin (10 μg), nalidixic acid (30 μg), amoxycillin (10 μg), ampicillin (10 μg), trimethoprim/sulfamethoxazole (1.25/23.75 μg), and tetracycline (30 μg). Zone diameters were measured (mm) and analyzed according to the Clinical & Laboratory Standards Institute (CLSI; CLSI, 2018) and European Committee on Antimicrobial Susceptibility Testing (EUCAST) (EUCAST, 2013) guidelines. Break points measured were recorded as susceptible or resistant, with isolates demonstrating intermediate resistance classified as susceptible, in order to avoid overestimation of resistance (Ta et al., 2014). Isolates with resistance to more than three antibiotic classes were classified as multidrug resistance (MDR).

Additionally*, E. coli* isolates were cultured in tryptone soy broth (Merck) at 37°C for 24 h, followed by genomic DNA extraction using the Quick gDNA Mini‐Prep Kit (Zymo Research) according to the manufacturer's instructions. The concentration of the DNA extracts was determined using the Qubit broad‐range double‐stranded DNA assay and the Qubit fluorometer (Life Technologies). For the detection of enterotoxigenic, enteropathogenic, enteroaggregative, enterohemorrhagic, enteroinvasive, and Shiga toxin virulence genes in the *E. coli* isolates, specific primers and primer concentrations as indicated in Table 1 were used for PCR reactions. *Escherichia coli* strains used as positive controls are indicated in Table 1, while ATCC 25922 (generic nonpathogenic *E. coli*) was used as a negative control. In addition, PCR‐grade water was used as the non‐template control. The PCR reactions (25 μL) contained between 100 ‐ 120 ng of the template DNA, the forward and reverse primer (Table 1), as well as 1× DreamTaq Green PCR Master Mix (Thermo Fisher Scientific). PCR cycling conditions were as follows: an initial denaturation at 95°C for 15 min, followed by 35 cycles of 94°C for 45 s, primer‐specific annealing temperature (Table 1) for 45 s, 68°C for 2 min, and a final extension for 7 min at 72°C. Products of the PCR reaction were electrophoresed on a 2% agarose gel (Thermo Fisher Scientific), prepared according to manufacturer's instructions at 120 V for 90 min, and thereafter, visualized using the GelDoc System (BioRad) in conjunction with Image Lab software (version 4.0.1).

**TABLE 1:** Genes that were screened for, primers used, and cycling conditions.

Gene	Primer type	Primer (5′–3′)^a^	Primer concentration (μM)	Positive controls	Primer‐specific annealing (°C)	Amplicon size (bp)	References
*stx 1*	Forward	ACA CTG GAT GAT CTC AGT GG	30	ATCC 35150	55	614	Omar and Barnard (2010)
	Reverse	CTG AAT CCC CCT CCA TTA TG					
*stx 2*	Forward	CCA TGA CAA CGG ACA GCA GTT	30	ATCC 35150	55	779	Omar and Barnard (2010)
	Reverse	CCT GTC AAC TGA GCA CTT TG					
*eaeA*	Forward	CTG AAC GGC GAT TAC GCG AA	60	ATCC 35150	55	917	Omar and Barnard (2010)
	Reverse	GAC GAT ACG ATC CAG					
*bfpA*	Forward	AAT GGT GCT TGC GCT TGC TGC	21	DSM 8703, DSM 8710	68	324	López‐Saucedo et al. (2003)
	Reverse	GCC GCT TTA TCC AAC CTG GTA					
*lt*	Forward	GGC GAC AGA TTA TAC CGT GC	40	DSM 10973	68	410	Pass et al. (2000)
	Reverse	CGG TCT CTA TAT TCC CTG TT		DSM 27503			
*st*	Forward	TTT CCC CTC TTT TAG TCA GTC AAC TG	20	DSM 10973	68	160	Pass et al. (2000
	Reverse	GGC AGG ATT ACA ACA AAG TTC ACA		DSM 27503			
pCVD4321AA probe	Forward	CTG GCG AAA GAC TGA ATC AT	30	DSM 27502	53	630	Schmidt et al. (1995)
	Reverse	CAA TGT ATA GAA ATC CGC TGT T					
*ipaH*	Forward	GTT CCT TGA CCG CCT TTC CGA TAC CGT C	42	DSM 9028, DSM 9034	60	600	Sethabutr et al. (1994)
	Reverse	GCC GGT CAG CCA CCC TCT GAG AGT AC					
*ial*	Forward	GGT ATG ATG ATG ATG ATG GGC	20	DSM 9028, DSM 9034	55	630	Cruz et al. (2014)
	Reverse	GGA GGC CAA CAA TTA TTT CC					

^a^ ATCC 25922 was used as a negative control and PCR‐grade water was additionally used as the nontemplate control.

### 2.4 Confirmation of extended‐spectrum β‐lactamase and AmpC production in presumptive extended spectrum β‐lactamase Enterobacteriaceae isolates

Forty‐four presumptive ESBL‐producing Enterobacteriaceae isolates including *E. coli* (*n* = 11), *Klebsiella* spp. (*n* = 18), and *Enterobacter* spp. (*n* = 15) were cultured as previously described in BHI and on Mueller–Hinton agar plates to screen for ESBL and AmpC production. The double‐disk synergy test using cefotaxime (30 μg), ceftazidime (30 μg), and cefpodoxime (10 μg) alone and in combination with clavulanic acid (10 μg) (Mast Diagnostics, Johannesburg) (EUCAST, 2013). The agar plates were incubated at 37°C for 24 h. Additionally, ESBL production in presumptive ESBL–*Enterobacter* spp. was confirmed using cefepime (30 μg) alone and in combination with clavulanic acid (10 μg) (Mast Diagnostics). The confirmation of AmpC production in all isolates was done using the AmpC detection set (Mast diagnostics). Zone diameters were measured (mm) and analyzed according to the CLSI (2018) and EUCAST (2013) guidelines.

### 2.5 Antimicrobial resistance screening of extended‐spectrum β‐lactamase‐ and AmpC‐producing Enterobacteriaceae isolates

Confirmed ESBL‐ and AmpC‐producing *E. coli* (*n* = 11), *Enterobacter* spp. (*n* = 13), and *Klebsiella* spp. (*n* = 17) isolates were then subjected to additional antimicrobial screening against amoxicillin (10 μg), ampicillin (10 μg), amoxicillin/clavulanic acid (20 μg/10 μg), cefoxitin (30 μg), cefepime (30 μg), cefpodoxime (10 μg), ceftazidime (30 μg), imipenem (10 μg), tetracycline (30 μg), neomycin (10 μg), gentamicin (10 μg), chloramphenicol (30 μg), cefotaxime (30 μg), ciprofloxacin (5 μg), and nitrofurantoin (300 μg). *Klebsiella pneumonia* ATCC 700603, *E. coli* NCTC 13315, *E. coli* ATCC 25922, and *E. cloacae* NCTC 1406 were used as positive and negative controls as described by the manufacturer (Mast Diagnostics). Zone diameters were measured, and results were recorded as previously described.

## 3 RESULTS

### 3.1 Coliform, *Escherichia coli*, Enterobacteriaceae, and *Staphylococcus aureus* counts

For fresh produce obtained from school gardens, cabbage samples had the highest mean Enterobacteriaceae and coliform counts at 5.12 log cfu g^−1^ and 4.46 log cfu g^−1^, respectively (Table 2). However, spinach had the highest mean *E. coli* counts (1.06 log cfu g^−1^) while mean *S. aureus* counts were highest in Chinese spinach samples (3.49 log cfu g^−1^; Table 2). Enterobacteriaceae, coliform, *E. coli*, and *S. aureus* counts for fresh produce obtained from school storerooms were highest in tomatoes (5.65 log cfu g^−1^), carrots (4.28 log cfu g^−1^), tomatoes (0.61 log cfu g^−1^), and onions (1.49 log cfu g^−1^), respectively (Table 3). Mean indicator organism counts for carrot samples obtained from the school storerooms were higher than for carrot samples obtained from the school gardens. Mean *E. coli* counts were the highest in spinach growing in the school gardens at 1.06 log cfu g^−1^ (Table 2). While the highest *S. aureus* counts were observed in Chinese spinach growing in the school gardens (Table 2). Apples in the storeroom contained the lowest *E. coli* counts for fresh produce in the storerooms, while the lowest *S. aureus* count was observed in carrots growing in the school gardens (Table 2). No *E. coli* were enumerated from the lettuce samples.

**TABLE 2:** Enterobacteriaceae, coliforms, *Escherichia coli*, and *Staphylococcus aureus* counts (log cfu/g) on fresh produce obtained from school gardens.

Fresh produce type (*n*)	Enterobacteriaceae		Coliforms		*Escherichia coli*		*Staphylococcus aureus*	
	Range, min–max^a^	Mean ± SD	Range, min–max	Mean ± SD	Range, min–max	Mean ± SD	Range, min–max	Mean ± SD
Cabbage (20)	0.00–6.90	5.12 ± 1.80	3.53–5.16	4.46 ± 0.49	0.00–3.00	0.80 ± 0.96	0.00–3.54	1.74 ± 1.16
Carrots (17)	3.59–5.69	4.74 ± 0.59	1.35–4.75	2.92 ± 1.04	0.00–1.079	0.26 ± 0.41	0.00–3.28	0.97 ± 1.08
Chinese spinach (13)	2.19–6.15	4.73 ± 1.26	1.64–5.01	3.55 ± 1.33	0.00–3.32	0.76 ± 1.21	2.00–5.02	3.49 ± 1.05
Lettuce (5)	4.12–5.10	4.62 ± 0.36	3.09–4.72	4.08 ± 0.60	0.00–0.00	0.00 ± 0.00	0.78–1.88	1.25 ± 0.38
Onions (27)	2.52–5.77	4.48 ± 1.00	0.00–5.39	3.63 ± 1.12	0.00–3.05	0.39 ± 0.95	0.00–3.60	1.44 ± 1.21
Spinach (110)	0.00–6.97	4.73 ± 1.17	0.00–5.97	3.89 ± 0.94	0.00–4.57	1.06 ± 1.27	0.00–4.51	2.34 ± 1.35

Abbreviation: SD, standard deviation.

^a^ The range indicates the minimum (min) and maximum (max) log cfu/g for each fresh produce type.

**TABLE 3:** Enterobacteriaceae, coliforms, *Escherichia coli*, and *Staphylococcus aureus* counts (log cfu/g) on fresh produce obtained from school storerooms.

Fresh produce type (*n*)	Enterobacteriaceae		Coliforms		*Escherichia coli*		*Staphylococcus aureus*	
	Range, min–max^a^	Mean ± SD	Range, min–max	Mean ± SD	Range, min–max	Mean ± SD	Range, min–max	Mean ± SD
Apples (27)	0.00–6.49	3.37 ± 1.66	0.00–5.62	2.46 ± 1.44	0.00–1.44	0.07 ± 0.28	0.00–3.08	0.98 ± 0.94
Cabbage (39)	0.00–6.81	5.02 ± 1.84	0.00–6.14	3.68 ± 1.75	0.00–3.60	0.50 ± 1.00	0.00–3.86	1.23 ± 1.30
Carrots (9)	4.73–6.63	5.38 ± 0.76	3.60–4.75	4.28 ± 0.35	0.00–2.85	0.50 ± 0.97	0.78–2.78	1.14 ± 0.81
Onions (42)	1.49–5.35	3.56 ± 1.13	1.20–4.88	3.04 ± 1.16	0.00–4.26	0.36 ± 0.94	0.00–2.95	1.49 ± 1.00
Tomatoes (12)	4.42–6.43	5.65 ± 0.68	0.00–4.85	3.11 ± 1.83	0.00–2.43	0.61 ± 0.91	0.00–2.70	1.47 ± 1.06

Abbreviation: SD, standard deviation.

^a^ The range indicates the minimum (min) and maximum (max) log cfu/g for each fresh produce type.

### 3.2 Detection of Enterobacteriaceae and *Listeria monocytogenes*

A total of 73 Enterobacteriaceae isolates were detected on fresh produce obtained from schools in the Gauteng and Mpumalanga Provinces. These included *E. coli* (*n* = 43; both generic and ESBL producing), *Enterobacter* spp. (*n* = 13), as well as *Klebsiella* spp. (*n* = 17). *Escherichia coli* was detected in 10.0% of cabbage (two of 20 samples), 11.8% carrots (two of 17 samples), and 11.1% Chinese spinach (two of 18 samples), respectively, as well as 3.7% onions (one of 27 samples) and 20.0% spinach (21 of 105 samples) obtained from the school gardens. From fresh produce obtained from the storerooms, *E. coli* was detected in 7.1% onions (three of 42 samples) and 16.7% tomatoes (two of 12 samples).

Of these *E. coli* isolates, 25.6% (11 of 43) of them were found to be ESBL and/or AmpC producing and were detected in 5.6% Chinese spinach (one of 18 samples), 7.4% onions (two of 27 samples), and 4.8% spinach (five of 105 samples) from the school gardens, whereas in the storerooms, they were detected from 4.8% onions (two of 42 samples), 8.3% tomatoes (one of 12 samples), as well as 22.2% carrots (two of 9 samples).

*Klebsiella pneumoniae* and *Klebsiella oxytoca* (*n* = 17) isolates were found to be ESBL and/or AmpC producing and were detected in 5.6% Chinese spinach (one of 18 samples), 11.1% onions (three of 27 samples), as well as 6.7% of spinach (seven of 105 samples) obtained from the school gardens. Extended‐spectrum β‐lactamase *Klebsiella* spp. was also detected from 11.9% onions (five of 42 samples), 22.2% carrots (two of nine samples), and 2.6% cabbages (one of 39 samples) from the school storerooms.

*Enterobacter* spp. (*n* = 13)‐producing ESBL was detected in 15% of cabbage (three of 20 samples), 11.1% of Chinese spinach (two of 18 samples), 3.7% of onions (one of 27 samples), 6.8% of spinach (seven of 105 samples), and 20% of lettuce (one of five samples) samples obtained from the school gardens. In the storerooms, ESBL–*Enterobacter* spp. were detected in 2.4% of onions (one of 42 samples), 3.7% of apples (one of 27 samples), 16.7% of tomatoes (two of 12 samples), and 7.7% of cabbage samples (three of 39 samples).

*Listeria monocytogenes* and *Salmonella* spp. were not detected in any of the fresh produce samples obtained from schools in the Gauteng and Mpumalanga Provinces.

### 3.3 Antimicrobial resistance and virulence gene screening of *Escherichia coli* isolates

No virulence genes were detected in the *E. coli* isolates that were screened. However, MDR was found in 60.5% of these 43 *E. coli* isolates (Table 4). *Escherichia coli* isolates (*n* = 43) displayed resistance to amoxicillin (62.8%), ampicillin (60.5%), trimethoprim (55.8), and tetracycline (55.8%). This was followed by resistance to cephalothin (51.2%), nitrofurantoin (46.5%), streptomycin (46.5%), nalidixic acid (46.5%), ciprofloxacin (44.2%), and cefotaxime (41.9%) with the least resistance to chloramphenicol (20.9%) and gentamicin (20.9%). Six (18.6%) *E. coli* isolates obtained from spinach collected from schools 9 and 10 and two *E. coli* isolates obtained from carrot samples collected from school 11 showed resistance to eight antibiotic classes (Table 4). Similarly, six of the *E. coli* isolates found on two spinach samples from schools 11 and 12, as well as on two onion samples from schools 7 and 8, and two tomato samples from schools 10 and 11 were resistant to seven classes of antibiotics. *Escherichia coli* isolates that were resistant to nine classes of antibiotics were found on one carrot sample and two spinach samples, all obtained from school 7.

**TABLE 4:** Antimicrobial resistance patterns of *Escherichia coli* found on fresh produce obtained from schools in the Gauteng and Mpumalanga Provinces.

Number of classes resistant to	Number (percentage) of isolates resistant to antibiotic classes	Most frequent antibiotic resistance pattern displayed by isolate (number of isolates)	Fresh produce types
0	10 (23.26)		Spinach (9), Chinese spinach (1)
1	3 (6.98)	^a^	Spinach (2), carrots (1)
2	3 (6.98)	CIP5C, S10C (2)	Spinach (3)
3	1 (2.33)	^a^	Carrots
4	3 (6.98)	A10C, AP10C, TS25C, T30C (2)	Spinach (2), cabbage (1)
5	2 (4.65)	KF30C, N300C, A10C, AP10C, TS25C, T30C (2)	Spinach (1), tomatoes (1)
6	4 (9.30)	KF30C, N300C, NA30C, A10C, AP10C, TS25C, T30C (4)	Cabbage (1), Carrots (1), tomatoes (1), spinach (1)
7	6 (13.95)	CTX30C, CIP5C, KF30C, S10C, A10C, AP10C, T30C (5)	Spinach (2), onions (2), tomatoes (2)
8	8 (18.60)	CTX30C, CIP5C, KF30C, N300C, S10C, A10C, AP10C, TS25C, T30C (8)	Spinach (6), carrots (2)
9	3 (6.98)	CTX30C, CIP5C, C30C, KF30C, GM10C, N300C, S10C, NA30C, A10C, AP10C, TS25C, T30C (3)	Spinach (2), carrots (1)

^a^ Antibiotic resistance patterns for isolates demonstrating resistance to the same number of classes were all different.

### 3.4 Antimicrobial resistance screening of extended‐spectrum β‐lactamase‐ and AmpC‐producing *Escherichia coli*, *Enterobacter*, and *Klebsiella* species

Of the 41 ESBL‐ and/or AmpC‐producing isolates, 47.8% were AmpC producers, 78.0% were ESBL producers, while 24.4% were both AmpC and ESBL producers. These included *E. coli* (*n* = 11), *Enterobacter* spp. (*n* = 13), *Klebsiella* spp. (*n* = 17). Of these 41 AmpC‐ and/or ESBL‐producing isolates, 97.6% were resistant to neomycin and nitrofurantoin followed by 95.1% of the isolates showing resistance to both ampicillin and amoxicillin. Resistance to tetracycline and trimethoprim was seen in 82.9% and 87.8% of the *E. coli* isolates, respectively, whereas resistance to ciprofloxacin and amoxicillin/clavulanic acid was seen in 78.1% of the isolates. Resistance to cefoxitin, gentamicin, and chloramphenicol was seen in 39.0%, 34.2%, and 22.0% of the isolates, respectively. Only 14.6% of the isolates were resistant to imipenem, an antibiotic belonging to the carbapenem class of antibiotics. Resistance against the third‐generation cephalosporins, cefotaxime, ceftazidime, and cefpodoxime was found seen in 78.1%, 82.9%, as well as 97.6% of ESBL‐ and/or AmpC‐producing Enterobacteriaceae. About 90.0% of these isolates were resistant to cefepime, a fourth‐generation cephalosporin. MDR was seen in 100% of the ESBL‐ and/or AmpC‐producing *Enterobacter*iaceae isolates, with up to 46.3% of these isolates resistant to eight classes of antibiotics (Table 5).

**TABLE 5:** Antimicrobial resistance patterns of extended‐spectrum β‐lactamase *Escherichia coli*, *Klebsiella* spp., and *Enterobacter* spp. found on fresh produce obtained from schools in the Gauteng and Mpumalanga Provinces.

Number of antibiotic classes	Number of isolates resistant to the number of antibiotic classes (%)	Most frequent antibiotic resistance pattern displayed by isolate (number of isolates)	Fresh produce types
4	1 (2.44)	^a^	Spinach
5	4 (9.76)	^a^	Spinach (1), cabbage (1), tomatoes (2)
7	10 (24.39)	AP10C, A10C, CPM30C, TS25C, T30C, NE10C, GM10C, CPD10C, CAZ30C, CTX30C, CIP5C, NI300C (2)	Spinach (5), onions (3), cabbage (1), carrots (1)
8	19 (46.34)	AP10C, A10C, AUG30C, CPM30C, TS25C, T30C, NE10C, CPD10C, CAZ30C, CTX30C, CIP5C, NI300C (4)	Spinach (8), onions (4), carrots (3), tomatoes (2), lettuce (1), cabbage (1)
9	7 (17.07)	AP10C, A10C, AUG30C, FOX30C, CPM30C, TS25C, T30C, NE10C, GM10C, C30C, CPD10C, CAZ30C, CTX30C, CIP5C, NI300C (3)	Spinach (6), cabbage (1)

^a^ Antibiotic resistance patterns for isolates demonstrating resistance to the same number of classes were all different.

## 4 DISCUSSION

Fresh produce is included in global and national school feeding menus in addition to the starch and protein component to ensure that learners get the required vitamins, minerals, and nutrients daily (Rendall‐Mkosi et al., 2013). Most vegetables are cooked and fruit such as apples, bananas, and oranges are served raw. However, the present study has shown that fresh produce grown and supplied to schools in the Gauteng and Mpumalanga Provinces are not always compliant with food safety criteria (based on previous SA Department of Health guidance, under review) (Department of Health, 2010) due to the presence of MDR *E. coli* and ESBL and/or AmpC‐producing Enterobacteriaceae as well as coliforms, *E. coli*, and *S. aureus*.

In this study, 86.0% and 31.0% of the fresh produce (from the school gardens and those delivered to the school) exceeded the coliform and *E. coli* guidelines, respectively, based on the previous Department of Health guidelines (Department of Health, 2010). Keeping in mind that fresh produce is grown on a smaller scale at schools and is mostly supplied to the school by independent suppliers based on the Department of Basic Education procurement processes (Rendall‐Mkosi et al., 2013).

Du Plessis et al. (2017) described mean coliform counts of 4.0 and 3.3 log cfu/g for cabbage samples obtained from vendors and retailers, respectively. These were comparable to the mean coliform counts observed from cabbage samples in this study, While the mean *E. coli* counts for spinach in this study did not exceed 1.1 log cfu/g, similar to those reported by Du Plessis et al. (2017; 0.8 and 0.4 log cfu/g). Similarly, an *E. coli* mean count of 0.7 log cfu/g for spinach was reported by Johnston et al. (2005), also lower than the mean *E. coli* count for spinach in the present study.

*Escherichia coli* (*n* = 43) isolates were detected on fresh produce samples from the garden and storeroom of the schools. Moreover, 20.0% of spinach samples indicated the presence of *E. coli* isolates in the present study. In a study by Jongman and Korsten (2016), *E. coli* was found in 18.0% of baby spinach, 20.0% of lettuce, and 27.0% of cabbage samples. The *E. coli* prevalence in spinach was similar to that of *E. coli* found in our study. However, no *E. coli* was found in the lettuce samples, while *E. coli* were found in 10% of cabbage samples in this study. In contrast to our study, *E. coli* was found in up to 73.3% and 100% of spinach samples as well as 3.3% and 6.7% of cabbage samples from retailers and street vendors, respectively, in SA (Du Plessis et al., 2017). These authors also found an *E. coli* prevalence of 8.3% on onion samples from a farm, which was higher than the 3.7% found in our study for onions obtained from the garden. For onions obtained from the storeroom, the prevalence of *E. coli* was 7.1%. Due to the general lack of cold room storage facilities at schools visited and subsequent results found in this study, it is considered important to assess the influence of storage on the microbiological quality of fresh produce in schools.

When compared to the SA Department of Health, Public Health England, and Hong Kong's Centre for Food Safety Microbiological Guidelines, levels of coliform, *E. coli*, *Enterobacter*iaceae, and *S. aureus* on fresh produce in this study were found unsatisfactory (Centre for Food Safety, Hong Kong, 2014; Department of Health, 2010; Public Health England, 2013). This highlights the importance of mitigation through proper washing and cooking (Bacon et al., 2003). Cooking may decrease the levels of bacteria in food (Wang et al., 2012). However, this does not apply to *S. aureus*, toxins (Bintsis, 2017). The bacteria may be susceptible to heat, but the toxins may survive and be able to cause disease (Bintsis, 2017). Cross‐contamination after cooking may also occur (Murray et al., 2017). Therefore, it is important that proper hygiene practices are followed to prevent foodborne diseases (Bacon et al., 2003). Not all fresh produce at the schools is washed and cooked before consumption. Apples were found not to harbor any pathogens in this study, and were the main fruit served at the schools visited. The washing of fresh produce with adequate sanitizers is also important in decreasing potential pathogen contamination (Gil et al., 2009; Olaimat & Holley, 2012). Allende et al. (2008) demonstrated in their study the need for wash water sanitizers to effectively eliminate pathogens in water. The schools visited did not use water sanitizers and relied on only using potable water to wash the apples (observation). However, potable water was not always available at these schools due to lack of resources or water cuts in their respective areas, further posing a challenge to maintaining adequate facilities and personal hygiene in food preparation facilities.

In contrast to the present study where *E. coli* isolates detected did not harbor the diarrheagenic virulence genes that were screened for, other studies have detected pathogenic *E. coli* in fresh produce. Castro‐Rosas et al. (2012) found *stx1*, *stx2*, and *ial* virulence genes in *E. coli* isolated from spinach, tomato, and lettuce, whereas du Plessis et al. (2015) were able to detect the *stx1* gene in *E. coli* detected on onions. Although no diarrheagenic virulence genes were detected in *E. coli* isolates in the present study, 60.4% and 62.8% of the *E. coli* isolates displayed resistance to ampicillin and amoxicillin, respectively. Furthermore, 55.8% of these *E. coli* isolates in our study were resistant to tetracycline and trimethoprim/sulfamethoxazole. Ampicillin resistance has also been reported in previous studies to be high among *E. coli*. Rasheed et al. (2014) found the dominant type of resistance to be ampicillin and amoxicillin, followed by tetracycline, cotrimoxazole, and streptomycin. Tetracycline resistance in this study was found to be 55.8%, higher than that reported by Faour‐Klingbeil et al. (2016) which was 42.0%.

MDR was seen in 67.0% of the *E. coli* isolates detected on fresh produce in the study carried out by Faour‐Klingbeil et al. (2016), similar to our study where 60.5% of the *E. coli* isolates were multidrug resistant. The most used antibiotics in animal production systems are tetracyclines, aminoglycosides, and penicillins (Kimera et al., 2020). Similarly, penicillins and tetracyclines, as well as sulfonamides (trimethoprim), are also widely used in the SA Public Health Sector (Schellack et al., 2017). *Escherichia coli* isolates in this study were mostly resistant to penicillins, trimethoprim, and tetracyclines, indicating that the widespread use of these antibiotics may be contributing to and may be leading to MDR development in bacterial pathogens. The implications for particularly immunocompromised people, who may be exposed to these resistant bacteria through fresh produce handling, are of concern due to obviously more limited treatment options (Schellack et al., 2017).

Our study also indicated that ESBL‐ and/or AmpC‐producing *E. coli*, *Enterobacter* spp., and *Klebsiella* spp. are present in fresh produce. Furthermore, these isolates were resistant to cefotaxime (78.1%), ceftazidime (82.1%), cefpodoxime (97.6%), third‐generation cephalosporins, as well as cefepime (90.2%), a fourth‐generation cephalosporin. Kim et al. (2015) and Zurfluh et al. (2015) reported 100% and 88.3% resistance to cefotaxime in ESBL‐producing *Enterobacter*iaceae isolates, higher than in our study. However, resistance to ceftazidime (15.8%) and cefepime (10.2%) was lower in the study by Kim et al. (2015). Resistance to non‐β‐lactam antibiotics was found in this study, with resistance to nitrofurans and aminoglycosides antibiotic classes being dominant. Similar to our study, Richter et al. (2019) also reported that 94.8% of Enterobacteriaceae isolates were resistant to the aminoglycoside class. A 100% resistance to ampicillin was reported by Mesbah Zekar et al. (2017). However, in our study, 95.1% of the ESBL‐ and/or AmpC‐producing isolates were resistant to ampicillin. In contrast to our study, 100% of ESBL‐producing Enterobacteriaceae isolates were susceptible to ampicillin.

Carbapenem resistance has come under the spotlight in SA as carbapenem‐resistant Enterobacteriaceae have caused outbreaks in hospitals (NICD, 2019; SAnews, 2020). Although these outbreaks were not related to food, these bacteria are able to genetically transfer their antimicrobial resistance to other related bacteria. The present study found carbapenem resistance in 14.6% of the ESBL‐ and/or AmpC‐producing Enterobacteriaceae, higher than the 0% and 10.6% resistance previously reported in similar studies (Kim et al., 2015; Singh et al., 2017). MDR was reported in 100% of the ESBL‐ and/or AmpC‐producing Enterobacteriaceae isolates in this study, whereas in other studies it was reported to be 96.1% (Richter et al., 2019) and 78.3% (Zurfluh et al., 2015). The CDC (2013) and WHO (2016) have described carbapenem‐resistant *Enterobacter*iaceae as a huge threat. These bacteria are resistant to almost all antibiotics and cause death in half of the patients infected with them. Therefore, antimicrobial resistance, moreover, carbapenem resistance in Enterobacteriaceae isolates found on fresh produce at schools is concerning.

Raw fresh produce samples obtained from surveyed schools in this study were found to not always comply with generally considered levels of coliform, *E. coli*, Enterobacteriaceae, as well as *S. aureus*. Thus, a need for a national improved food safety strategy is needed to prevent foodborne disease outbreaks at schools and to better monitor produced and procured fresh produce. Forthcoming studies should focus on investigating the implementation of good food safety management principles at schools to ensure food is safe for consumption. Future studies should also seek to determine the potential link between the microbiological quality of fresh produce grown and served at schools to the production and handling practices. The training of food handlers at these schools is imperative and should be conducted on a regular basis. Similarly, the state of food safety at schools should also be monitored and audited as part of a food safety assurance system. Additionally, quantitative microbial risk assessment studies should be done to determine the risk involved when school children are exposed to certain foods provided through the school feeding scheme or sold in or near school premises. We envisage that the results of this study will be considered by international and national governments to develop new policies and guidelines that will help to safeguard the safety of food provided in the national school feeding program.

## AUTHOR CONTRIBUTIONS

**Thabang Msimango:** Data curation (equal); investigation (equal); writing – original draft (equal). **Stacey Duvenage:** Conceptualization (equal); methodology (lead); project administration (lead); supervision (supporting); writing – review and editing (equal). **Erika Du Plessis:** Conceptualization (equal); project administration (equal); supervision (supporting); writing – review and editing (supporting). **Lise Korsten:** Conceptualization (equal); funding acquisition (lead); supervision (lead); writing – review and editing (equal).

## CONFLICT OF INTEREST STATEMENT

The authors declare no conflict of interest.

## Data Availability

The data that support the findings of this study are available from the corresponding author upon reasonable request.
